# Breast cancer survivors’ perspectives on a clinical decision tool to support individualized exercise prescriptions and discussions

**DOI:** 10.1007/s00520-026-10358-x

**Published:** 2026-01-29

**Authors:** Oliver W. A. Wilson, Kaitlyn M. Wojcik, Eleanor M. Kerr, Ilse Rivera, Emma Tian, Jacob D. Schneider, Rachelle Brick, David Berrigan, Kosuke Tamura, Laura Q. Rogers, Wendy Demark-Wahnefried, Richard L. Street, Jinani Jayasekera

**Affiliations:** 1 Decision Sciences Research Laboratory, National Institute on Minority Health and Health Disparities, Intramural Research Program, National Institutes of Health, Bethesda, MD USA; 2 Ripple Effect Communications, Inc., Rockville, MD USA; 3 Health Systems and Interventions Research Branch of the Healthcare Delivery Research Program in the Division of Cancer Control & Population Sciences at the National Cancer Institute, National Institutes of Health,, Rockville, MD USA; 4 Division of Cancer Control &, Population Sciences at the National Cancer Institute, National Institutes of Health, Rockville, MD USA; 5 Division of General Internal Medicine and Population Science, Department of Medicine at University of Alabama Birmingham, Birmingham, AL USA; 6 Department of Nutrition Sciences at, University of Alabama Birmingham, Birmingham, AL USA; 7 Department of Communication and Journalism at, Texas AandM University, College Station, TX USA

**Keywords:** Breast cancer patients, Physical activity, Clinical decision support systems, Exercise prescription, Exercise Discussion, Exercise referral

## Abstract

**Purpose:**

To examine breast cancer survivors’ perspectives on the prototype (paper-draft) of a clinical decision tool to support individualized exercise prescriptions and discussions within clinical settings.

**Methods:**

A sample of ≥90 female breast cancer survivors aged ≥35 years across the U.S. were recruited to complete an online survey that collected data on survivors’ characteristics and perspectives of a prototype of the tool. Survey items were adapted from validated pre-existing instruments and refined via cognitive interviews.

**Results:**

Ninety-eight of the 142 individuals screened were deemed eligible and completed the survey. Most breast cancer survivors agreed that a tool would be useful (84.7%) and increase confidence to discuss exercise with a healthcare provider (74.5%). Among tool uses, the highest agreement was found for education (84.1%) and encouragement to exercise (79.3%). Improving the ability to do everyday tasks (74.0%), quality-of-life (72.6%), and energy (71.4%) were rated as the top exercise benefits to include in a tool. Agreement on tool usefulness, uses, and inputs varied by survivors’ demographic, clinical, and contextual characteristics. For example, agreement that a tool would increase their confidence to discuss exercise was lower among younger survivors (<50 years, 67.7%) than older survivors (≥50 years, 78.5%).

**Conclusions:**

An evidence-based clinical decision tool offering individualized exercise information could support exercise prescriptions and discussions within clinical settings. However, a one-size-fits-all tool may not address existing disparities in exercise participation. Further research among underserved women is needed.

**Supplementary Information:**

The online version contains supplementary material available at 10.1007/s00520-026-10358-x.

## Introduction

Physical activity offers breast cancer survivors a variety of potential benefits, including a reduction in their risk of recurrence, morbidity (e.g., cardiovascular disease risk), and mortality [1, 2]. A common form of physical activity is exercise, defined as planned structured physical activity performed with the intention of improving or maintaining physical fitness [3]. However, exercise participation often declines after breast cancer diagnosis and treatment among women [4–6]. National-level data show that a low proportion of breast cancer survivors in the U.S. meet exercise recommendations (aerobic [≥150min/week] = 37.7%], and muscle-strengthening [≥2days/week] = 17.6%) [7]. As a result, many breast cancer survivors in the U.S. are not fully experiencing the potential benefits of exercise.

Conversations about exercise with healthcare providers can meaningfully increase patient exercise [8, 9]. Contemporary cancer exercise guidelines recommend breast cancer survivors receive individualized exercise prescriptions from healthcare and exercise professionals [10–12]. In fact, discussions about exercise are a component of current cancer survivorship plans [13], and are now required for national accreditation of breast cancer centers [14]. However, only 54.4% of breast cancer survivors in the U.S. report discussing exercise with a healthcare provider [15], and just 52.3% report receiving exercise advice [16]. Health literacy may constrain survivors’ participation in exercise discussions [17, 18]. Whereas limited knowledge, time, and self-efficacy are among the main constraints to healthcare providers discussing exercise in clinical settings [19, 20].

Disparities in both patient-provider communication about exercise and actual exercise participation among women diagnosed with breast cancer are evident in the U.S. A recent study reported a lower likelihood of receiving exercise advice among breast cancer survivors with less than a high school education or who have comorbidities compared to survivors with at least a college degree or no comorbidities, respectively [16]. Racial, ethnic [7], socioeconomic [7, 21], and geographic [22, 23] disparities in exercise participation have also been reported among survivors. Further, exercise participation among breast cancer survivors may be influenced by individuals’ clinical (e.g., stage at diagnosis) [24–26], physical (e.g., pain), psychosocial (e.g., social support), and environmental (e.g., recreational amenity access) characteristics [27–29].

Clinical decision tools or decision support systems may address the barriers to exercise discussions by offering timely exercise information that is tailored to a survivor’s demographic, clinical, and contextual characteristics [30]. Such tools may help shared decision-making through improvements in knowledge and decisional self-efficacy [31]. Tools would also help healthcare providers meet survivorship plan standards by facilitating exercise discussions. Several tools have been developed to help healthcare providers to assess cancer survivors’ exercise, offer exercise advice and recommendations, and even refer survivors to rehabilitation and/or exercise services [32–36]. However, a recent review did not identify any evidence-based tools to support individualized exercise prescriptions that consider the breast cancer survivors’ individual demographic, clinical (e.g. stage of diagnosis, treatment history), and contextual characteristics [30]. In this study, we aimed to evaluate breast survivors’ perspectives on a prototype of a clinical decision tool designed to support individualized exercise prescriptions and discussions for breast cancer within clinical settings.

## Methods

### Development of the survey and prototype (paper-draft) clinical tool

Survey items were adapted from pre-existing instruments when possible (Supplemental Table 1). Cognitive interviews were conducted with five survivors and ten healthcare providers (i.e., breast oncologists, primary care physicians, occupational therapists, and exercise specialists) to assess their understanding of the survey questions. A Spanish version of the survey was developed and tested for translation accuracy and comprehension by two Spanish-speaking researchers. The survey was revised to incorporate the feedback from interviews. Agreement was measured using a five-point Likert scale ranging from strongly disagree (1) to strongly agree (5).

The development of the prototype of the tool is described elsewhere [37]. Briefly, tool development was informed by the Collaborative Deliberation Model [38], which outlines five components to be considered during tool development (Constructive Engagement, Choice Recognition, Learning, Preference Elicitation, and Integration). The prototype of the tool was developed with input from breast cancer survivors (*n*=5) and the authorship team that included a tool developer (*n*=1), exercise specialists (*n*=4), and clinicians (*n*=3). The tool consisted of a section for inputs such as individual (e.g., disability), clinical (e.g., treatment history), and contextual (e.g., education) determinants of exercise. These inputs were used to generate individualized estimates for potential benefits of exercise in breast cancer outcomes (e.g., survival) using an established mathematical model [39–42]. We also included components in the tool to note any clinical conditions a healthcare provider should consider during an exercise discussion (e.g., neuropathy) (Supplemental Table 1).

The paper-draft was updated to develop a ‘wireframe’ for the prototype of the tool using survivor perspectives. The ‘wireframe’ for this study was a pdf file that illustrated the layout, functionality, and content hierarchy for the tool. Changes made to improve comprehension and understanding included offering further details on clinical characteristics, such as stage and hormone receptor type, as well as conditions caused by breast cancer and cancer treatments. For example, survivors recommended including ‘weakness, numbness, and pain’ alongside neuropathy to enhance understanding of the term. Changes were also made to the Spanish translation of the tool, such as changing ‘recidiva’ to ‘recurrencia’ for the translation of recurrence.

### Participants and recruitment

Women previously diagnosed with breast cancer aged ≥35 years residing in the U.S. were purposively recruited via an external contractor. At least 10 Alaska Native, American Indian, Black or African American, Middle Eastern or North African (MENA), Native Hawaiian, and White participants were purposively recruited. In addition, at least 10 English-speaking and at least 10 Spanish-speaking Hispanic participants were recruited. Spanish-speaking participants completed the Spanish survey. Screening questions were used to exclude those who did not meet inclusion criteria. Participants received $75 for cognitive interviews conducted to develop the survey (described below) and $30 for complete survey responses. Different participants were recruited to participate in the interview and survey. The study was approved by the National Institutes of Health Institutional Review Board and was considered as exempt based on the use of de-identified data. Informed consent was obtained from each participant.

### Measures

#### Demographic characteristics

Participants reported their age at the time of the survey (years), race, ethnicity, sexual orientation, disability [43, 44], and participation in aerobic (min/week) and muscle-strengthening exercise (days/week) in a typical week [45]. Participants had to respond to a screening question confirming that they were female to proceed to the survey.

#### Clinical characteristics

Other relevant information collected included the year of diagnosis, stage of diagnosis, hormone receptor status, treatment, and survivorship stage (pre-treatment, on-treatment, post-treatment, palliative care). In addition, women reported if they had ever experienced ataxia, bone metastases, extreme fatigue, lymphedema, peripheral neuropathy, recurrence, and/or second primary cancers [46–48].

#### Contextual characteristics

Data on U.S. state of residence, urbanicity (i.e., urban vs. rural), relationship status, education, household income, living arrangement (e.g., homeowner vs. nonhomeowner), and employment were gathered.

#### Clinical decision tool for individualized exercise prescriptions

Study participants were provided with an overview of a prototype (paper-draft) of a tool that could provide individualized exercise recommendations considering their demographic, clinical, and contextual characteristics (Supplemental Figure 1). Women rated their agreement on the overall usefulness of the tool and if its use would increase their confidence to talk about exercise with their healthcare provider(s). Women rated their agreement on whether the tool could be used to: (1) educate themselves on the benefits and risks of exercise, (2) encourage them to exercise, (3) facilitate shared decision-making about exercise with a healthcare provider(s), (4) identify resources within the healthcare system to support exercise, and (5) obtain a referral to an exercise professional. Participants rated their agreement on whether potential tool inputs (i.e., clinical characteristics), benefits (e.g., life expectancy), and conditions associated with cancer and treatment (e.g., lymphedema) should be integrated into the tool. Feedback on suggested changes to make the tool more useful, increase confidence, and other inputs that should be included were collected via open-ended questions.

### Analyses

Descriptive statistics were computed using Microsoft Excel [49]. Agreement items were dichotomized into those who indicated agreement (i.e., agreed or strongly agreed) and those who did not (i.e., disagreed, or had a neutral response). This enabled reporting of overall percentages of agreement for tool usefulness, use, and input items Differences in tool usefulness, use, and input items based on survivor’s individual, clinical, and contextual characteristics were compared by computing stratified statistics.

Themes in the responses to open-ended questions were identified via thematic analysis [50]. Two researchers (OW and KW) independently analyzed qualitative data, revising interpretation using the constant comparison method [51]. Disagreements were resolved via consensus discussion and triangulation.

## Results

### Survivor characteristics

We aimed to recruit ≥90 breast cancer survivors via purposive sampling. Of the 142 women who were screened, 98 (69.0%) met the inclusion criteria (i.e., were a female breast cancer survivor, aged ≥35 years, currently residing in U.S.) and proceeded to complete the survey (Supplemental Table 2). The mean age of the sample was 47.2 years (standard deviation (SD)=12.2). Racial groups were represented relatively equally across the sample, and 22.4% were Hispanic. Over 80% were diagnosed with stage I-III cancer (81.6%) and were either undergoing or post-treatment (92.9%). Ataxia, bone metastases, extreme fatigue, lymphedema, and/or peripheral neuropathy were reported by most participants (79.6%). The percentage of survivors meeting aerobic and muscle-strengthening exercise guidelines was 77.4% and 80.3%, respectively. Most identified as straight (89.5%), were residents of urban/suburban areas (86.7%), were homeowners (55.1%), were in a relationship (67.7%), worked full-time (60.2%), and had a disability (54.1%). Regarding education and income, the largest groups among participants were those with a college degree (42.9%) and reporting a household income ≥$75,000 (45.9%).

### Overall findings

#### Tool usefulness

Most participants agreed that a tool would be useful (84.7%; 95% confidence interval (CI):76.9–92.4%) and would increase confidence to discuss exercise (74.5%; 95% CI:64.5–84.5%). Among the 47 responses to the open-ended question on how the tool would make them more confident to discuss exercise with their healthcare provider(s), the themes identified were increased knowledge (*n*=18, 38.3%), encouragement/comfort (*n*=15, 31.9%), discussion/question ideas (*n*=11, 23.4%), and tracking (*n*=3, 6.4%).

#### Tool uses

Most would use a tool to educate themselves on the benefits and risks of exercise (84.1%; 95%CI:75.5–92.8%), encourage themselves to exercise (79.3%; 95%CI:69.4–89.1%), be referred to an exercise professional (76.5%; 95%CI:66.0–87.1%), identify resources within the healthcare system to support themselves to exercise (72.0%; 95%CI:60.5–83.4%), and facilitate shared decision-making between themselves and their healthcare provider(s) about exercise (68.3%; 95%CI:56.1–80.5%). Over half mentioned encouragement, accountability, and/or motivation (*n*=36, 55.4%) in response to the open-text question concerning how a tool would support their exercise. Other prevailing responses included offering individualized information (*n*=13, 20.0%), access to general information (*n*=12, 18.5%), and tracking (*n*=12, 12.3%).

#### Benefits and conditions

Survivors rated items on ‘exercise benefits’ and various ‘conditions associated with cancer and treatment’ that should be integrated into a tool. For benefits, over 70% agreed that the ability to do everyday tasks (74.0%), quality-of-life (72.6%), energy (71.4%), bone health (71.1%), sleep (71.1%), and recurrence (70.8%) should be integrated (Supplemental Table 3). Cognitive difficulty (75.5%) was the only condition with agreement ≥70% (Supplemental Table 4).

#### Demographic, clinical, and contextual inputs

Women also rated which demographic, clinical, and contextual characteristics should be included as inputs in a tool (Supplemental Table 5). Age (72.2%), readiness to exercise (73.5%), and access to exercise resources at home (79.6%) were the items with highest agreement. Conversely, agreement was lower than 50% on race and ethnicity (43.6%), utilities (41.8%), and residential greenness (41.2%). Agreement that race and ethnicity should be included was low across racial and ethnic groups. The main additional inputs mentioned in response to the open-text question were tool usability/accessibility and social support (Supplemental Table 6).

### Exploratory sub-group analyses

#### Demographic characteristics

Older (≥50 years) (84.6%) and younger (<50 years) (87.1%) women expressed similarly high levels of agreement that a tool would be useful. However, agreement that a tool would increase their confidence to discuss exercise was lower among younger survivors (67.7% vs. 78.5%). Agreement on tool uses was ≥10% higher among older vs. younger survivors. For example, 85.2% of older survivors agreed they would use a tool for shared decision-making compared to 60.0% of younger survivors. For contextual inputs, only childcare had lower agreement for older survivors (48.4% vs. 73.8%). Whereas phone access had higher agreement (83.9% (older) vs. 36.9% (younger)).

Although ≥80% agreed that a tool would be useful overall, agreement was lower among Alaska Native (72.7%) and MENA (54.5%) survivors. Agreement that a tool would increase confidence to discuss exercise varied across racial and ethnic groups with 50.0% among Asian to 90.0% among Black women. Agreement on tool uses also differed based on race and ethnicity.

#### Clinical characteristics

Agreement that a tool would increase confidence to discuss exercise was higher among those diagnosed with early stage, hormone receptor positive, human epidermal growth factor receptor 2 (HER2) negative breast cancer (i.e., less advanced/less aggressive breast cancer) compared to those with later stage, hormone receptor negative, HER2-positive cancer.

#### Contextual characteristics

Though ≥80% of urban, suburban, and rural survivors agreed that a tool would be useful, agreement that a tool would increase confidence to discuss exercise was higher among suburban survivors (83.7%) compared to urban (69.0%) and rural survivors (63.6%). By contrast, rural survivors reported greater agreement (90.0%) that they would use a tool to obtain a referral compared to suburban (81.6%), and urban (66.7%) survivors. Survivors in a relationship had greater agreement for access to home exercise resources (87.7% vs. 63.3%) and lower agreement for childcare (60.0% vs. 80.0%) and finances (60.8% vs. 70.0%). Women with a baccalaureate degree or higher tended to report greater agreement for most inputs compared to women with some college education who, in turn, tended to report greater agreement than women with a high school education or lower.

## Discussion

Contemporary cancer exercise guidelines recommend providing individualized exercise prescriptions [10–12]. A variety of approaches (e.g., the Moving Through Cancer initiative [32], decision algorithms [i.e., models] such as Exercise in Cancer Evaluation and Decision Support [EXCEEDS] [33–35], and Cancer Exercise application [36]) have been developed to support offering exercise prescriptions to or triage to services for cancer survivors. However, to our knowledge, there are no evidence-based clinical support systems capable of generating individualized outcomes (e.g., risk of breast cancer death) associated with physical activity for breast cancer survivors considering their individual characteristics [30].

Overall, breast cancer survivors’ perspectives on a tool that could offer individualized exercise recommendations to support exercise-related decisions in clinical settings were positive. Education and encouragement were the highest rated tool uses, indicating that a tool may help to address gaps in knowledge and support relating to exercise participation among breast cancer survivors [27, 52–54]. Women also indicated that they would use a tool to receive exercise referrals. Therefore, a tool may help increase the frequency and uptake of exercise referrals, which are persistent issues in cancer survivor exercise promotion [55, 56].

Most women indicated that they would use a tool to support shared decision-making with their healthcare provider regarding exercise. Shared decision-making is an important part of cancer survivors developing survivorship care plans in collaboration with their healthcare providers [57], and is associated with breast cancer survivor exercise participation [58]. Thus, findings align with those of previous studies that found breast cancer survivors want exercise to be included and better addressed a part of survivorship care plans [59–61]. Further refinements may be required to facilitate shared decision-making about exercise in clinical settings. For example, providing survivors with individualized exercise information generated by a tool prior to clinical consultations may help survivors to deliberate and be aware of their options under less time pressure [62–64]. Different modes of communication could also be considered. For instance, a pre-visit evidence-based educational video has been shown to increase knowledge and decrease distress among breast cancer survivors while stimulating thoughts and questions about their care [65].

Breast cancer survivors rated age, readiness to exercise, and access to exercise resources at home as the most important tool inputs. Studies have shown that behavioral change theories are useful when promoting exercise among breast cancer survivors [66–68]. Our finding that survivors viewed exercise readiness as an essential input reinforces the importance of considering behavioral change theories when developing a tool and accompanying messages to promote exercise, especially since readiness to exercise is closely aligned with constructs from behavioral change theories such as self-efficacy, motivation, intention, and attitude [69–74].

The importance of access to home resources highlights the need for healthcare professionals to consider the personal preferences and resources at the disposal of survivors when providing exercise recommendations. For example, exercise preferences with respect to the physical location (e.g., home vs. healthcare setting), supervision (i.e., supervised vs. unsupervised), and the social environment (i.e., alone vs. with company) can vary among survivors [75–82]. However, some survivors may not have home exercise resources and/or the financial means to participate in supervised exercise. A tool that considers the resources available to survivors in addition to their clinical needs and preferences could provide healthcare providers an opportunity to individualize care based on survivor’s needs and preferences. For instance, women diagnosed with more advanced cancer could be referred to a lower intensity supervised exercise program, whereas women with earlier stage cancer may receive information and resources to support home based exercise or a referral to community exercise programs. Better consideration of women’s exercise preferences may improve women’s perspectives on the tool, as well as their uptake of provider advice and referrals.

It is worth noting that ‘race and ethnicity’ was among the lowest rated inputs. Some have advocated that race be included in clinical decision tools when it is directly linked with racism and contextual factors [83]. However, race-based medicine has a problematic history in the U.S [84]. Further work is needed to establish whether, and if so how, race and ethnicity should be included in a tool that offers individualized exercise information for breast cancer survivors, particularly in light of the ongoing debate, combined with recent evidence that healthcare algorithms could mitigate, perpetuate, or exacerbate racial/ethnic disparities irrespective of the explicit use of race or ethnicity [85].

Shorter-term outcomes such as the ability to do everyday tasks, quality-of-life, energy, and sleep rated among the top benefits. Though many of these ‘benefits’ are the inverse of the ‘conditions’ associated with breast cancer and related treatment that were examined, the outcomes were rated higher as benefits compared to conditions. The opposite was true for cognition, which was lowest rated benefit (55.2%) but the highest rated condition (75.5%). Further work is warranted to determine survivors’ preferences regarding of gain or loss framing for each outcome. For example, many breast cancer survivors experience sleep distirbances [86, 87]. Whether a sleep-related outcome should be framed in terms of ‘improved sleep’ and/or ‘less likely to experience sleep disturbances or insomnia’ is worthy of investigation in future research.

We assessed the potential differences in tool usefulness, uses, and inputs based on breast cancer survivors’ demographic, clinical, and contextual characteristics. The differences revealed highlight the need for individualized exercise recommendations for breast cancer survivors [10–12], and may offer insight to inform the development of a tool to address existing disparities in exercise communication and participation. For example, women diagnosed with later-stage or more aggressive forms of breast cancer perceived the tool less favorably. These findings suggest that the tool needs to be updated to consider the unique exercise preferences of these women [76, 77, 88] and support them in overcoming barriers they may encounter such as, financial toxicity (i.e., financial problems related to the cost of medical care) [89] and impairments specifically related to cancer and treatments [82, 90]. The perspectives of women who participate in less physical activity, are older, and are less affluent are also needed as the sample recruited was not representative of U.S. breast cancer survivors.

The lower ratings of shared decision-making compared with other tool uses may be indicative of a level of medical mistrust or uncertainty regarding the motives clinicians or healthcare institutions [91–93]. Studies have shown lack of trust in the healthcare system may vary based on race, ethnicity, and education among breast cancer survivors [92, 93]. Health literacy may also influence patient-healthcare provider communication and shared decision-making [94, 95]. Further research is warranted to understand whether, and if so how, a tool could address medical mistrust and health literacy to support patient-provider communication, especially among historically underserved and underrepresented populations. For instance, translation of information into women’s preferred language may mitigate health literacy barriers related to language [96]. Culturally-tailored exercise education and information may also help mitigate the effects of medical mistrust and health literacy [97, 98].

The benefits with the greatest agreement for inclusion in a tool were the ability to do everyday tasks, quality-of-life, and energy. Breast cancer survivors who initiate exercise are likely to experience these exercise-related benefits [10, 99], which in turn may improve their motivation, efficacy, and intention to habitually adhere to exercise long-term [100]. Thus, a tool providing individualized information about the exercise benefits for which breast cancer survivors have an interest and are likely to experience has the potential to meaningfully increase exercise participation, and ultimately, health.

Our study has several limitations. The women in our study were younger, more affluent, and more physically active compared to the wider population of breast cancer survivors in the U.S. This is indicative of self-selection bias and may limit generalizability and explain some of findings. For example, inputs concerning women’s physical and functional impairments were rated low, despite such impairments being frequently reported as barriers to exercise by older breast cancer survivors and/or women diagnosed with advanced breast cancer [27, 28, 82]. As such, items concerning physical and functional may still need to be included in a tool for it to meet the needs of the wider population of breast cancer survivors. The modest sample size constrained our ability to rigorously examine differences in perspectives based on demographic, clinical, and contextual characteristics. Reliance on self-reported measures also means responses were susceptible to social desirability bias [101]. However, our sampling strategy ensured the inclusion of the perspectives of women who have historically been excluded from cancer research [102, 103]. We did not examine specific exercise participation preferences (e.g., intensity, setting), insurance coverage for exercise, or willingness to pay out-of-pocket of and/or travel to participate in exercise. Future research is warranted to examine how these factors could be incorporated into a tool. Finally, given the time constraints experienced in clinical settings, establishing how many inputs can feasibly be included in a tool is a crucial next step.

### Future directions

Future work should seek to acquire insight into the perspectives of a more representative sample of breast cancer survivors. In particular, survivors who are less physically active, older, have lower socioeconomic status, and reside in rural communities. Further research is also needed to understand how to best communicate exercise information generated by the tool to survivors. The performance of the tool in comparison to usual care and other methods of offering exercise prescriptions to breast cancer survivors will also need to be tested.

Our intention is to continue to iteratively develop this clinical decision tool with input from survivors and healthcare providers. The perspectives of healthcare providers (i.e., breast oncologists, primary care physicians, exercise specialists, occupational therapists, physical therapists, advanced care providers, nurses, patient navigators, and social workers) on the same clinical decision tool are reported in a separate paper [37]. The wireframe will be revised based on the findings reported in this formative research before being revised again following future cognitive interviews with healthcare providers and more representative breast cancer survivors. The revised wireframe will then be translated into a functional prototype web-based clinical decision tool, which will be tested for feasibility and acceptability. The version of the tool that is tested will incorporate items that elucidate patient preferences concerning exercise participation (e.g., exercise frequency, intensity, type; willingness to pay and/or travel for exercise) and communication (i.e., how survivors would prefer to receive exercise information and from whom) using a question prompt list currently being developed. Ultimately, a Beta-version tool will be used to conduct a clinical trial that will evaluate the effects of individualized exercise prescriptions on exercise self-efficacy, exercise intentions, and exercise participation of breast cancer survivors.

## Conclusions

In summary, our findings indicate an evidence-based tool that offers breast cancer survivors individualized exercise information could support exercise discussions and prescriptions within clinical settings. Proximal benefits (e.g., improved ability to do everyday tasks) appear to be the most important to include in a tool from survivors’ perspectives. Differences in agreement on tool usefulness, uses, and inputs based on demographic, clinical, and contextual characteristics suggest that a generic, one-size-fits-all tool, or approach may not address existing exercise participation disparities. Our study also reinforces the need for further research to support the development of individualized clinical decision tools for use in breast cancer care settings. Particularly research that examines the perspectives of underserved and underrepresented women and those who participate in minimal exercise.

## Supplementary Information

Below is the link to the electronic supplementary material.

Supplementary file1 (DOCX 387 KB)

## Data Availability

The datasets during and/or analyzed during the current study are available from the corresponding author on reasonable request.

## Declarations

### Disclaimer

This research was supported [in part] by the Intramural Research Program of the NIH, NIMHD. The contributions of the NIH author(s) were made as part of their official duties as NIH federal employees, are in compliance with agency policy requirements, and are considered Works of the United States Government. However, the findings and conclusions presented in this paper are those of the author(s) and do not necessarily reflect the views of the NIH or the U.S. Department of Health and Human Services. The study funders had no role in the design of the study, the collection, analysis, or interpretation of the data, the writing of the manuscript, or the decision to submit the manuscript for publication.

## References

[1] Wilson OWA, Wojcik KM, Cohen CM et al (2025) Exercise and cardiovascular health among breast cancer survivors: A scoping review of current observational evidence. Cardiooncology 11(1):24. 10.1186/s40959-025-00310-z 40012001 10.1186/s40959-025-00310-z PMC11863919

[2] Wilson OWA, Matthews CE, Wojcik KM et al (2025) The effects of post-diagnosis recreational aerobic exercise among breast cancer survivors: a systematic review/meta-analyses. Cancer Epidemiol Biomarkers Prev 34(8):1252–1263. 10.1158/1055-9965.EPI-24-1798 40387563 10.1158/1055-9965.EPI-24-1798 PMC12314521

[3] Caspersen CJ, Powell KE, Christenson GM (1985) Physical activity, exercise, and physical fitness: definitions and distinctions for health-related research. Public Health Rep 100(2):126–131 3920711 PMC1424733

[4] Kwan ML, Sternfeld B, Ergas IJ et al (2012) Change in physical activity during active treatment in a prospective study of breast cancer survivors. Breast Cancer Res Treat 131(2):679–690. 10.1007/s10549-011-1788-4 21953007 10.1007/s10549-011-1788-4 PMC3273453

[5] Irwin ML, Crumley D, McTiernan A et al (2003) Physical activity levels before and after a diagnosis of breast carcinoma: The Health, Eating, Activity, and Lifestyle (HEAL) study. Cancer 97(7):1746–1757 12655532 10.1002/cncr.11227 PMC3034406

[6] Irwin ML, McTiernan A, Bernstein L, Gilliland FD, Baumgartner R, Baumgartner K, Ballard-Barbash R (2004) Physical activity levels among breast cancer survivors. Med Sci Sports Exerc 36(9):1484 15354027 PMC3000611

[7] Wojcik KM, Wilson OWA, Sheils MS, Sheppard VL, Jayasekera J (2024) Racial, ethnic, and socioeconomic disparities in meeting physical activity guidelines among female breast cancer survivors in the United States. Cancer Epidemiol Biomark Prev 33(12):1610–1622. 10.1158/1055-9965.EPI-24-0650 10.1158/1055-9965.EPI-24-0650 PMC11609821 39269270

[8] Orrow G, Kinmonth A-L, Sanderson S, Sutton S (2012) Effectiveness of physical activity promotion based in primary care: Systematic review and meta-analysis of randomised controlled trials. BMJ 344:e1389. 10.1136/bmj.e1389 22451477 10.1136/bmj.e1389 PMC3312793

[9] Wilson OWA, Wojcik KM, Rogers LQ et al (2025) Exercise communication for breast cancer survivors: a systematic scoping review. JAMA Netw Open 8(5):e258862. 10.1001/jamanetworkopen.2025.8862 40377942 10.1001/jamanetworkopen.2025.8862 PMC12084847

[10] Campbell KL, Winters-Stone KM, Wiskemann J et al (2019) Exercise guidelines for cancer survivors: consensus statement from international multidisciplinary roundtable. Med Sci Sports Exerc 51(11):2375–2390. 10.1249/mss.0000000000002116 31626055 10.1249/MSS.0000000000002116 PMC8576825

[11] Physical Activity Guidelines Advisory Committee (2018) 2018 Physical activity guidelines advisory committee scientific report. Washington, DC; U.S. Department of Health and Human Services. https://odphp.health.gov/sites/default/files/2019-09/PAG_Advisory_Committee_Report.pdf

[12] Ligibel JA, Bohlke K, May AM et al (2022) Exercise, diet, and weight management during cancer treatment: ASCO guideline. J Clin Oncol 40(22):2491–2507. 10.1200/jco.22.00687 35576506 10.1200/JCO.22.00687

[13] American Cancer Society. ASCO Cancer Treatment and Survivorship Care Plans. Jul 1, 2024. https://www.cancer.org/cancer/survivorship/long-term-health-concerns/survivorship-care-plans.html. Accessed 1 Jul 2024

[14] American College of Surgeons. Optimal Resources for Breast Cancer: 2024 Standards. 2023. https://accreditation.facs.org/accreditationdocuments/NAPBC/Standards/Optimal_Resources_for_Breast_Care_2024.pdf. Accessed 15 Aug 2024

[15] Siembida EJ, Kent EE, Bellizzi KM, Smith AW (2020) Healthcare providers’ discussions of physical activity with older survivors of cancer: potential missed opportunities for health promotion. J Geriatr Oncol 11(3):437–443. 10.1016/j.jgo.2019.05.007 31122873 10.1016/j.jgo.2019.05.007

[16] Wojcik KM, Wilson OWA, Kamil D, Rajagopal PS, Schonberg MA, Jayasekera J (2024) Clinical and sociodemographic determinants of older breast cancer survivors’ reports of receiving advice about exercise. Breast Cancer Res Treat. 10.1007/s10549-024-07460-1 39347888 10.1007/s10549-024-07460-1 PMC11522097

[17] Tang W, Li Z, Tang C, Wang X, Wang H (2017) Health literacy and functional exercise adherence in postoperative breast cancer patients. Patient Prefer Adherence. 11(null):781-786. 10.2147/PPA.S127925 10.2147/PPA.S127925 PMC5402901 28458522

[18] Plummer LC, Chalmers KA (2017) Health literacy and physical activity in women diagnosed with breast cancer. Psychooncology 26(10):1478–1483. 10.1002/pon.4318 27859877 10.1002/pon.4318

[19] Fong AJ, Faulkner G, Jones JM, Sabiston CM (2018) A qualitative analysis of oncology clinicians’ perceptions and barriers for physical activity counseling in breast cancer survivors. Support Care Cancer 26(9):3117–3126. 10.1007/s00520-018-4163-8 29574619 10.1007/s00520-018-4163-8

[20] Smaradottir A, Smith AL, Borgert AJ, Oettel KR (2017) Are we on the same page? Patient and provider perceptions about exercise in cancer care: a focus group study. J Natl Compr Canc Netw 15(5):588–594. 10.6004/jnccn.2017.0061 28476738 10.6004/jnccn.2017.0061

[21] Paxton RJ, Phillips KL, Jones LA, Chang S, Taylor WC, Courneya KS, Pierce JP (2012) Associations among physical activity, body mass index, and health-related quality of life by race/ethnicity in a diverse sample of breast cancer survivors. Cancer 118(16):4024–4031. 10.1002/cncr.27389 22252966 10.1002/cncr.27389 PMC3330155

[22] Olson EA, Mullen SP, Rogers LQ, Courneya KS, Verhulst S, McAuley E (2014) Meeting physical activity guidelines in rural breast cancer survivors. Am J Health Behav 38(6):890–899. 10.5993/ajhb.38.6.11 25341266 10.5993/ajhb.38.6.11 PMC4209408

[23] Rogers LQ, Markwell SJ, Courneya KS, McAuley E, Verhulst S (2011) Physical activity type and intensity among rural breast cancer survivors: patterns and associations with fatigue and depressive symptoms. J Cancer Surviv 5(1):54–61. 10.1007/s11764-010-0160-8 21110134 10.1007/s11764-010-0160-8 PMC3041842

[24] Smith SA, Ansa BE, Yoo W, Whitehead MS, Coughlin SS (2018) Determinants of adherence to physical activity guidelines among overweight and obese African American breast cancer survivors: implications for an intervention approach. Ethn Health 23(2):194–206. 10.1080/13557858.2016.1256376 27838922 10.1080/13557858.2016.1256376 PMC5429994

[25] Coletta AM, Marquez G, Thomas P et al (2019) Clinical factors associated with adherence to aerobic and resistance physical activity guidelines among cancer prevention patients and survivors. PLoS ONE 14(8):e0220814. 10.1371/journal.pone.0220814 31369653 10.1371/journal.pone.0220814 PMC6675393

[26] Troeschel AN, Leach CR, Shuval K, Stein KD, Patel AV (2018) Physical activity in cancer survivors during “re-entry" following cancer treatment. Prev Chronic Dis 15:E65. 10.5888/pcd15.170277 10.5888/pcd15.170277 PMC5985854 29806579

[27] Michael M, Goble J, Hawk M, Kujawa DJ (2021) Reported barriers impeding adherence to a physical exercise program in patients with breast cancer: a systematic review. Rehabil Oncol. 10.1097/01.REO.0000000000000220

[28] Lavallée JF, Abdin S, Faulkner J, Husted M (2019) Barriers and facilitators to participating in physical activity for adults with breast cancer receiving adjuvant treatment: a qualitative metasynthesis. Psychooncology 28(3):468–476. 10.1002/pon.4980 30657225 10.1002/pon.4980

[29] Wurz A, St-Aubin A, Brunet J (2015) Breast cancer survivors’ barriers and motives for participating in a group-based physical activity program offered in the community. Support Care Cancer 23(8):2407–2416. 10.1007/s00520-014-2596-2 25605568 10.1007/s00520-014-2596-2 PMC4483247

[30] Wojcik KM, Kamil D, Zhang J et al (2024) A scoping review of web-based, interactive, personalized decision-making tools available to support breast cancer treatment and survivorship care. J Cancer Surviv. 10.1007/s11764-024-01567-6 38538922 10.1007/s11764-024-01567-6 PMC11436482

[31] Bailey RA, Pfeifer M, Shillington AC, Harshaw Q, Funnell MM, VanWingen J, Col N (2016) Effect of a patient decision aid (PDA) for type 2 diabetes on knowledge, decisional self-efficacy, and decisional conflict. BMC Health Serv Res 16(1):10. 10.1186/s12913-016-1262-4 26762150 10.1186/s12913-016-1262-4 PMC4712511

[32] Schmitz KH, Campbell AM, Stuiver MM et al (2019) Exercise is medicine in oncology: engaging clinicians to help patients move through cancer. CA Cancer J Clin 69(6):468–484. 10.3322/caac.21579 31617590 10.3322/caac.21579 PMC7896280

[33] Covington KR, Marshall T, Campbell G et al (2021) Development of the exercise in cancer evaluation and decision support (EXCEEDS) algorithm. Support Care Cancer 29(11):6469–6480. 10.1007/s00520-021-06208-7 33900458 10.1007/s00520-021-06208-7 PMC8525648

[34] Wood KC, Pergolotti M, Marshall T et al (2022) Usability, acceptability, and implementation strategies for the exercise in cancer evaluation and decision support (EXCEEDS) algorithm: a Delphi study. Support Care Cancer 30(9):7407–7418. 10.1007/s00520-022-07164-6 35614154 10.1007/s00520-022-07164-6

[35] Stout NL, Brown JC, Schwartz AL et al (2020) An exercise oncology clinical pathway: Screening and referral for personalized interventions. Cancer 126(12):2750–2758. 10.1002/cncr.32860 32212338 10.1002/cncr.32860 PMC7258139

[36] Schwartz AL, Helling W, Griffin S, Mikuls S, Weber B (2024) Cancer exercise app design: tailored exercise for people living with and beyond cancer. Physical Activity, Exercise Cancer 1(1):14–22. 10.61486/ZQOS1684

[37] Jayasekera J, Wilson OWA, Wojcik KM et al (2025) Healthcare provider perspectives on a clinical decision tool to support individualized exercise prescriptions and discussions for breast cancer survivors. J Cancer Surviv. 10.1007/s11764-025-01750-3 10.1007/s11764-025-01750-3 PMC12310178 40074972

[38] Elwyn G, Lloyd A, May C et al (2014) Collaborative deliberation: a model for patient care. Patient Educ Couns 97(2):158–164. 10.1016/j.pec.2014.07.027 25175366 10.1016/j.pec.2014.07.027

[39] Jayasekera J, Sparano JA, O’Neill S et al (2021) Development and validation of a simulation model-based clinical decision tool: identifying patients where 21-gene recurrence score testing may change decisions. J Clin Oncol 39(26):2893–2902. 10.1200/jco.21.00651 34251881 10.1200/JCO.21.00651 PMC8425835

[40] Jayasekera J, Stein S, Wilson OWA et al (2024) Benefits and harms of mammography screening in 75 + women to inform shared decision-making: a simulation modeling study. J Gen Intern Med 39(3):428–439. 10.1007/s11606-023-08518-4 38010458 10.1007/s11606-023-08518-4 PMC10897118

[41] Jayasekera J, Zhao A, Schechter C et al (2023) Reassessing the benefits and harms of risk-reducing medication considering the persistent risk of breast cancer mortality in estrogen receptor–positive breast cancer. J Clin Oncol 41(4):859–870. 10.1200/jco.22.01342 36455167 10.1200/JCO.22.01342 PMC9901948

[42] Jayasekera JC, Wilson OWA, Schechter C et al (2025) Development and validation of a simulation model–based tool to support individualized physical activity discussions and prescriptions for breast cancer survivors. JCO Clin Cancer Inform. 9:e2500151. 10.1200/CCI-25-00151 41105911 10.1200/CCI-25-00151 PMC12543000

[43] National Library of Medicine. National Institutes of Health Common Data Elements Repository. https://cde.nlm.nih.gov/home

[44] Powers SL, Pitas NA, Mowen AJ (2023) Critical consciousness of systemic racism in parks among park agency directors and policymakers: An environmental justice tool for recreation and conservation leaders. Soc Nat Resour 1-24. 10.1080/08941920.2023.2250737

[45] National Cancer Insititute. Health Information National Trends Survey. https://hints.cancer.gov/view-questions/all-hints-questions.aspx. Accessed 28 June 28 2024

[46] National Cancer Insititute. Office of Cancer Survivorship Definitions. https://cancercontrol.cancer.gov/ocs/definitions#terms

[47] Sheikh-Wu SF, Anglade D, Downs CA (2023) A cancer survivorship model for holistic cancer care and research. Can Oncol Nurs J 33(1):4–16. 10.5737/236880763314 36789222 10.5737/236880763314 PMC9894370

[48] National Cancer Insititute. Palliative Care in Cancer. https://www.cancer.gov/about-cancer/advanced-cancer/care-choices/palliative-care-fact-sheet. Accessed 17 Sept 2024

[49] Microsoft Corp (2025) Microsoft Excel (Version 16.105)

[50] Braun V, Clarke V (2006) Using thematic analysis in psychology. Qual Res Psychol 3(2):77–101. 10.1191/1478088706qp063oa

[51] Corbin J, Strauss A (2014) Basics of qualitative research: Techniques and procedures for developing grounded theory. 4th ed. Sage. Thousand Oaks, CA

[52] Browall M, Mijwel S, Rundqvist H, Wengström Y (2016) Physical activity during and after adjuvant treatment for breast cancer: An integrative review of women’s experiences. Integr Cancer Ther 17(1):16–30. 10.1177/1534735416683807 28008778 10.1177/1534735416683807 PMC5950941

[53] Buja A, Rabensteiner A, Sperotto M et al (2020) Health literacy and physical activity: a systematic review. J Phys Act Health 17(12):1259–1274. 10.1123/jpah.2020-0161 33129198 10.1123/jpah.2020-0161

[54] Cho J, Jung S-Y, Lee JE, et al (2014) A review of breast cancer survivorship issues from survivors' perspectives. J Breast Cancer 17(3):189-199. 10.4048/jbc.2014.17.3 10.4048/jbc.2014.17.3.189 PMC4197348 25320616

[55] Yang DD, Hausien O, Aqeel M, Klonis A, Foster J, Renshaw D, Thomas R (2017) Physical activity levels and barriers to exercise referral among patients with cancer. Patient Educ Couns 100(7):1402–1407. 10.1016/j.pec.2017.01.019 28189469 10.1016/j.pec.2017.01.019

[56] Ezenwankwo EF, Nnate DA, Usoro GD et al (2022) A scoping review examining the integration of exercise services in clinical oncology settings. BMC Health Serv Res 22(1):236. 10.1186/s12913-022-07598-y 35189864 10.1186/s12913-022-07598-y PMC8859567

[57] Ke Y, Zhou H, Chan RJ, Chan A. (2024) Decision aids for cancer survivors’ engagement with survivorship care services after primary treatment: A systematic review. J Cancer Surviv. 2024/04/01 18(2):288-317. 10.1007/s11764-022-01230-y 10.1007/s11764-022-01230-y PMC10960885 35798994

[58] Lim JW, Baik OM, Ashing-Giwa KT (2012) Cultural health beliefs and health behaviors in Asian American breast cancer survivors: a mixed-methods approach. Oncol Nurs Forum 39(4):388–397. 10.1188/12.Onf.388-397 22750897 10.1188/12.ONF.388-397

[59] Krok-Schoen JL, Naughton MJ, Noonan AM, Pisegna J, DeSalvo J, Lustberg MB (2020) Perspectives of survivorship care plans among older breast cancer survivors: a pilot study. Cancer Control 27(1):1073274820917208. 10.1177/1073274820917208 32233798 10.1177/1073274820917208 PMC7143997

[60] Burke NJ, Napoles TM, Banks PJ, Orenstein FS, Luce JA, Joseph G (2016) Survivorship care plan information needs: perspectives of safety-net breast cancer patients. PLoS ONE 11(12):e0168383. 10.1371/journal.pone.0168383 27992491 10.1371/journal.pone.0168383 PMC5161365

[61] Burg MA, Lopez ED, Dailey A, Keller ME, Prendergast B (2009) The potential of survivorship care plans in primary care follow-up of minority breast cancer patients. J Gen Intern Med 24 Suppl 2(Suppl 2):S467-71. 10.1007/s11606-009-1012-y 10.1007/s11606-009-1012-y PMC2763149 19838852

[62] Bomhof-Roordink H, Gärtner FR, Stiggelbout AM, Pieterse AH (2019) Key components of shared decision making models: a systematic review. BMJ Open 9(12):e031763. 10.1136/bmjopen-2019-031763 31852700 10.1136/bmjopen-2019-031763 PMC6937101

[63] Légaré F, Ratté S, Gravel K, Graham ID (2008) Barriers and facilitators to implementing shared decision-making in clinical practice: update of a systematic review of health professionals’ perceptions. Patient Educ Couns 73(3):526–535. 10.1016/j.pec.2008.07.018 18752915 10.1016/j.pec.2008.07.018

[64] Wieringa TH, León-García M, Espinoza Suárez NR et al (2024) The role of time in involving patients with cancer in treatment decision making: a scoping review. Patient Educ Couns 125:108285. 10.1016/j.pec.2024.108285 38701622 10.1016/j.pec.2024.108285

[65] Serpico V, Liepert AE, Boucher K, Fouts DL, Anderson L, Pell J, Neumayer L (2016) The effect of previsit education in breast cancer patients: A study of a shared-decision-making tool. Am Surg 82(3):259–265. 10.1177/000313481608200320 27099063

[66] Liu MG, Davis GM, Kilbreath SL, Yee J (2022) Physical activity interventions using behaviour change theories for women with breast cancer: a systematic review and meta-analysis. J Cancer Surviv 16(5):1127–1148. 10.1007/s11764-021-01104-9 34491527 10.1007/s11764-021-01104-9

[67] Bluethmann SM, Bartholomew LK, Murphy CC, Vernon SW (2017) Use of theory in behavior change interventions. Health Educ Behav 44(2):245–253. 10.1177/1090198116647712 27226430 10.1177/1090198116647712 PMC5503486

[68] Loprinzi PD, Cardinal BJ, Si Q, Bennett JA, Winters-Stone KM (2012) Theory-based predictors of follow-up exercise behavior after a supervised exercise intervention in older breast cancer survivors. Support Care Cancer 20(10):2511–2521. 10.1007/s00520-011-1360-0 22252545 10.1007/s00520-011-1360-0 PMC4010587

[69] Teixeira PJ, Carraça EV, Markland D, Silva MN, Ryan RM (2012) Exercise, physical activity, and self-determination theory: a systematic review. Int J Behav Nutr Phys Act 9(1):78. 10.1186/1479-5868-9-78 22726453 10.1186/1479-5868-9-78 PMC3441783

[70] Ryan RM, Deci EL (2000) Self-determination theory and the facilitation of intrinsic motivation, social development, and well-being. Am Psychol 44(1):68–78. 10.1037/0003-066X.55.1.68 10.1037//0003-066x.55.1.68 11392867

[71] Bandura A (2004) Health promotion by social cognitive means. Health Educ Behav 31(2):143–164. 10.1177/1090198104263660 15090118 10.1177/1090198104263660

[72] Bandura A (1977) Self-efficacy: toward a unifying theory of behavioral change. Psychol Rev 84(2):191–215 847061 10.1037//0033-295x.84.2.191

[73] Prochaska JO, Velicer WF (1997) The transtheoretical model of health behavior change. Am J Health Promot 12(1):38–48. 10.4278/0890-1171-12.1.38 10170434 10.4278/0890-1171-12.1.38

[74] Ajzen I (1991) The theory of planned behavior. Organ Behav Hum Decis Process 50(2):179–211. 10.1016/0749-5978(91)90020-T

[75] Forbes CC, Blanchard CM, Mummery WK, Courneya KS (2015) A comparison of physical activity preferences among breast, prostate, and colorectal cancer survivors in Nova Scotia, Canada. J Phys Act Health 12(6):823–833. 10.1123/jpah.2014-0119 25155470 10.1123/jpah.2014-0119

[76] Sweegers MG, Depenbusch J, Aaronson NK et al (2024) Metastatic breast cancer patients’ preferences for exercise programs: a latent class analysis using data from a survey in five European countries. Support Care Cancer 33(1):39. 10.1007/s00520-024-09068-z 39694910 10.1007/s00520-024-09068-z

[77] Sweegers MG, Depenbusch J, Kampshoff CS et al (2023) Perspectives of patients with metastatic breast cancer on physical exercise programs: results from a survey in five European countries. Support Care Cancer 31(12):694. 10.1007/s00520-023-08124-4 37955790 10.1007/s00520-023-08124-4 PMC10643348

[78] Borsati A, Toniolo L, Trestini I et al (2024) Feasibility of a novel exercise program for patients with breast cancer offering different modalities and based on patient preference. Eur J Oncol Nurs 70:102554. 10.1016/j.ejon.2024.102554 38615512 10.1016/j.ejon.2024.102554

[79] Rogers LQ, Markwell SJ, Verhulst S, McAuley E, Courneya KS (2009) Rural breast cancer survivors: exercise preferences and their determinants. Psychooncology 18(4):412–421. 10.1002/pon.1497 19241491 10.1002/pon.1497

[80] Rogers LQ, Courneya KS, Shah P, Dunnington G, Hopkins-Price P (2007) Exercise stage of change, barriers, expectations, values and preferences among breast cancer patients during treatment: a pilot study. Eur J Cancer Care 16(1):55–66. 10.1111/j.1365-2354.2006.00705.x 10.1111/j.1365-2354.2006.00705.x 17227354

[81] Schleicher E, McAuley E, Courneya KS et al (2023) Breast cancer survivors’ exercise preferences change during an exercise intervention are associated with post-intervention physical activity. J Cancer Surviv. 10.1007/s11764-023-01389-y 37120460 10.1007/s11764-023-01389-y PMC11572959

[82] Ten Tusscher MR, Groen WG, Geleijn E et al (2019) Physical problems, functional limitations, and preferences for physical therapist-guided exercise programs among Dutch patients with metastatic breast cancer: a mixed methods study. Support Care Cancer 27(8):3061–3070. 10.1007/s00520-018-4619-x 30610432 10.1007/s00520-018-4619-x

[83] Yearby R (2021) Race based medicine, colorblind disease: How racism in medicine harms us all. Am J Bioeth 21(2):19–27. 10.1080/15265161.2020.1851811 33280534 10.1080/15265161.2020.1851811

[84] Wright JL, Davis WS, Joseph MM, Ellison AM, Heard-Garris NJ, Johnson TL, AAP Board Committee on Equity (2022) Eliminating race-based medicine. Pediatrics. 10.1542/peds.2022-057998 35491483 10.1542/peds.2022-057998

[85] Siddique SM, Tipton K, Leas B et al (2024) The impact of health care algorithms on racial and ethnic disparities. Ann Intern Med 177(4):484–496. 10.7326/M23-2960 38467001 10.7326/M23-2960

[86] Costa AR, Fontes F, Pereira S, Gonçalves M, Azevedo A, Lunet N (2014) Impact of breast cancer treatments on sleep disturbances – a systematic review. Breast 23(6):697–709. 10.1016/j.breast.2014.09.003 25307946 10.1016/j.breast.2014.09.003

[87] Leysen L, Lahousse A, Nijs J et al (2019) Prevalence and risk factors of sleep disturbances in breast cancersurvivors: systematic review and meta-analyses. Support Care Cancer 27(12):4401–4433. 10.1007/s00520-019-04936-5 31346744 10.1007/s00520-019-04936-5

[88] Delrieu L, Vallance JK, Morelle M et al (2020) Physical activity preferences before and after participation in a 6-month physical activity intervention among women with metastatic breast cancer. Eur J Cancer Care 29(1):e13169. 10.1111/ecc.13169 10.1111/ecc.13169 31571315

[89] Ehsan AN, Wu CA, Minasian A et al (2023) Financial toxicity among patients with breast cancer worldwide: A systematic review and meta-analysis. JAMA Netw Open 6(2):e2255388. 10.1001/jamanetworkopen.2022.55388 36753274 10.1001/jamanetworkopen.2022.55388 PMC9909501

[90] Pinto M, Calafiore D, Piccirillo MC, Costa M, Taskiran OO, de Sire A (2022) Breast cancer survivorship: the role of rehabilitation according to the International Classification of Functioning Disability and Health—a scoping review. Curr Oncol Rep 24(9):1163–1175. 10.1007/s11912-022-01262-8 35403973 10.1007/s11912-022-01262-8 PMC9467947

[91] Berrigan D, Dean D Jr, Senft Everson N, D’Angelo H, Boyd P, Klein WMP, Han PKJ (2023) Uncertainty: A neglected determinant of health behavior? Front Psychol 14:1145879. 10.3389/fpsyg.2023.1145879 37251060 10.3389/fpsyg.2023.1145879 PMC10213393

[92] Mouslim MC, Johnson RM, Dean LT (2020) Healthcare system distrust and the breast cancer continuum of care. Breast Cancer Res Treat 180(1):33–44. 10.1007/s10549-020-05538-0 31983018 10.1007/s10549-020-05538-0 PMC7675785

[93] Sutton AL, He J, Edmonds MC, Sheppard VB (2019) Medical mistrust in Black breast cancer patients: acknowledging the roles of the trustor and the trustee. J Cancer Educ 34(3):600–607. 10.1007/s13187-018-1347-3 29552705 10.1007/s13187-018-1347-3 PMC7061268

[94] Sudore RL, Landefeld CS, Pérez-Stable EJ, Bibbins-Domingo K, Williams BA, Schillinger D (2009) Unraveling the relationship between literacy, language proficiency, and patient–physician communication. Patient Educ Couns 75(3):398–402. 10.1016/j.pec.2009.02.019 19442478 10.1016/j.pec.2009.02.019 PMC4068007

[95] Pérez-Stable EJ, El-Toukhy S (2018) Communicating with diverse patients: how patient and clinician factors affect disparities. Patient Educ Couns 101(12):2186–2194. 10.1016/j.pec.2018.08.021 30146407 10.1016/j.pec.2018.08.021 PMC6417094

[96] Gunn CM, Paasche-Orlow MK, Bak S et al (2020) Health literacy, language, and cancer-related needs in the first 6 months after a breast cancer diagnosis. JCO Oncol Pract 16(8):e741–e750. 10.1200/JOP.19.00526 32216715 10.1200/JOP.19.00526 PMC7427419

[97] Ashing K, Serrano M, Weitzel J, Lai L, Paz B, Vargas R (2014) Towards developing a bilingual treatment summary and survivorship care plan responsive to Spanish language preferred breast cancer survivors. J Cancer Surviv 8(4):580–594. 10.1007/s11764-014-0363-5 24859132 10.1007/s11764-014-0363-5

[98] Mendoza-Vasconez AS, Linke S, Muñoz M et al (2016) Promoting physical activity among underserved populations. Transl J Am Coll Sports Med. 10.1249/TJX.0000000000000014 10.1249/JSR.0000000000000276 PMC5371027 27399827

[99] Aune D, Markozannes G, Abar L et al (2022) Physical activity and health-related quality of life in women with breast cancer: A meta-analysis. JNCI Cancer Spectr 6(6) 10.1093/jncics/pkac072 10.1093/jncics/pkac072 PMC9727071 36474321

[100] Husebø AML, Dyrstad SM, Søreide JA, Bru E (2013) Predicting exercise adherence in cancer patients and survivors: a systematic review and meta-analysis of motivational and behavioural factors. J Clin Nurs 22(1–2):4–21. 10.1111/j.1365-2702.2012.04322.x 23163239 10.1111/j.1365-2702.2012.04322.x

[101] Fisher RJ (1993) Social desirability bias and the validity of indirect questioning. Article. J Consum Res 20(2):303–315

[102] Javier-DesLoges J, Nelson TJ, Murphy JD et al (2022) Disparities and trends in the participation of minorities, women, and the elderly in breast, colorectal, lung, and prostate cancer clinical trials. Cancer 128(4):770–777. 10.1002/cncr.33991 34806168 10.1002/cncr.33991

[103] Duma N, Vera Aguilera J, Paludo J et al (2018) Representation of minorities and women in oncology clinical trials: review of the past 14 years. J Oncol Pract 14(1):e1–e10. 10.1200/JOP.2017.025288 29099678 10.1200/JOP.2017.025288

