# Correction to: Breast cancer survivors’ perspectives on a clinical decision tool to support individualized exercise prescriptions and discussions

**DOI:** 10.1007/s00520-026-10899-1

**Published:** 2026-06-16

**Authors:** Oliver W. A. Wilson, Kaitlyn M. Wojcik, Eleanor M. Kerr, Ilse Rivera, Emma Tian, Jacob D. Schneider, Rachelle Brick, David Berrigan, Kosuke Tamura, Laura Q. Rogers, Wendy Demark‑Wahnefried, Richard L. Street, Jinani Jayasekera

**Affiliations:** 1 Decision Sciences Research Laboratory, National Institute on Minority Health and Health Disparities, Intramural Research Program, National Institutes of Health, Bethesda, MD USA; 2 Ripple Effect Communications, Inc., Rockville, MD USA; 3 Health Systems and Interventions Research, Healthcare Delivery Research Program, Division of Cancer Control & Population Sciences, National Cancer Institute, National Institutes of Health, Rockville, MD USA; 4 Division of Cancer Control & Population Sciences, National Cancer Institute, National Institutes of Health, Rockville, MD USA; 5 Division of General Internal Medicine and Population Science, Department of Medicine at University of Alabama Birmingham, Birmingham, AL USA; 6 Department of Nutrition Sciences, University of Alabama Birmingham, Birmingham, AL USA; 7 Department of Communication and Journalism, Texas A&M University, College Station, TX USA

**Correction to** **:** **Supportive Care in Cancer (2026) 34:139**

10.1007/s00520-026-10358-x

There are typesetting errors in the 'Results' section. Specifically, extra numbers have been erroneously printed next to the upper limit of each confidence interval.

In the "Tool Usefulness" section. The extra numbers appended to the confidence intervals should be removed as follows:

As published: (84.7%; 95% CI:76.9–92.4.9.4%) and (74.5%; 95% CI:64.5–84.5.5.5%)

As corrected: (84.7%; 95% CI:76.9–92.4%) and (74.5%; 95% CI:64.5–84.5%)

In the "Tool Uses" section,

As published: Most would use a tool to educate themselves on the benefits and risks of exercise (84.1%; 95%CI:75.5–92.8**.5.8**%), encourage themselves to exercise (79.3%; 95%CI:69.4–89.1**.4.1**%), be referred to an exercise professional (76.5%; 95%CI:66.0–87.1**.0.1**%), identify resources within the healthcare system to support themselves to exercise (72.0%; 95%CI:60.5–83.4**.5.4**%), and facilitate shared decision-making between themselves and their healthcare provider(s) about exercise (68.3%; 95%CI:56.1–80.5**.1.5**%).

As corrected: Most would use a tool to educate themselves on the benefits and risks of exercise (84.1%; 95%CI:75.5–92.8%), encourage themselves to exercise (79.3%; 95%CI:69.4–89.1%), be referred to an exercise professional (76.5%; 95%CI:66.0–87.1%), identify resources within the healthcare system to support themselves to exercise (72.0%; 95%CI:60.5–83.4%), and facilitate shared decision-making between themselves and their healthcare provider(s) about exercise (68.3%; 95%CI:56.1–80.5%).

The publisher regrets this error.

The original article has been corrected.

