# Supplementary material for: Breast cancer survivors’ perspectives on a clinical decision tool to support individualized exercise prescriptions and discussions
Source: Support Care Cancer. 2026 Jan 29;34(2):139. doi: 10.1007/s00520-026-10358-x (PMC12852301; doi:10.1007/s00520-026-10358-x)
Supplement: Supplementary file 1 — Supplementary file1 (DOCX 387 KB) [file 520_2026_10358_MOESM1_ESM.docx]

Supplemental Table 1. Survey question sources

| **Measure** | **Categories** | | **Source** |
| --- | --- | --- | --- |
| Individual characteristics | - Age - Race - Ethnicity - Sexual orientation | - Disability status - Aerobic exercise (min/week) - Muscle-strengthening exercise (days/week) | NIH CDE repository^42^;  Health Information National Trends Survey (HINTS)^44^ |
| Clinical characteristics | - Diagnosed with other cancer - Treatment history - Survivorship stage - Stage of diagnosis | - Hormone receptor status - HER2 status - Conditions | Cancer Exercise Guidelines^10^;  Expert input |
| Contextual characteristics | - Living arrangements - Urbanicity/rurality - Education | - Household income - Employment - State | NIH CDE repository^42^;  Powers et al.^43^ |
| Tool Inputs | - Age - Race and ethnicity - Stage at diagnosis - Tumor characteristics - Current treatment (e.g., chemotherapy, radiotherapy, endocrine therapy, surgery) - Historical treatments - Current comorbidities - Physical impairments (e.g., mobility, reaching) - Functional impairments (e.g., challenges with daily living) - Cognitive impairments (e.g., changes in memory or attention, difficulty problem solving) - Psychological impairments (e.g., changes in mood, feeling down, etc.) - Patient personal preferences (e.g., what activities they enjoy doing) - Readiness to exercise - Childcare (child supervision) - Clothing (availability of appropriate clothing and shoes) | - Finances (problems with insurance or debt) - Food (access to healthy food) - Housing (availability of safe and secure housing) - Internet/broadband (availability of internet to check email and schedule exercise sessions) - Phone (e.g., availability of a phone to connect with peer and health care professional) - Transportation (availability of transportation) - Utilities (whether an individual had utility companies cut off service due to not paying bills) - Residential greenness (availability of green spaces) - Neighborhood safety (safety of local environment) - Access to a facility or exercise program in the neighborhood or at work - Access to exercise resources at home - Other characteristics (open-text box) | NIH CDE repository^42^;  Cognitive Interviews |
| Individualized Benefits | - Improved quality of life - More likely to stay cancer recurrence free - Less likely to die of breast cancer - Less likely to die of cardiovascular disease - Less likely to die of all causes, - Improved life expectancy - Less tiredness / more energy - Improved cognition - Greater ability to do everyday tasks | - Less likely to experience anxiety - Less likely to experience depressive symptoms - Less likely to experience lymphedema - Improved bone health - Improved sleep - Less likely to be admitted to the hospital - Less like to experience adverse side effects during treatment - ‘Other’ (open-text box) | Cancer Exercise Guidelines^10^;  Cognitive Interviews |
| Conditions associated with cancer and cancer treatment | - Cognitive difficulty (mental slowing) - Neuropathy (weakness, numbness, and pain) - Sarcopenia/muscle weakness - Slowing and fatigue - Bone loss - Bone metastases - Lymphedema (swelling due to build-up of lymph fluid in the body) | - Symptom clusters (symptoms that occur at the same time) - Arthritis/musculoskeletal issues - Ataxia (problems with coordination, balance, walking) - Severe nutritional deficiencies (e.g., low calcium) - Cardiopulmonary disease - Nausea or diarrhea - Sexual dysfunction - ‘Other’ (open-text box) | Cancer Exercise Guidelines^10^;  Cognitive Interviews |

Notes. NIH = National Institutes of Health; CDE = Common Data Elements; HER2 = human epidermal growth factor receptor 2

**Supplemental Figure 1: Screenshots of the prototype (paper-draft) tool shown to survivors**


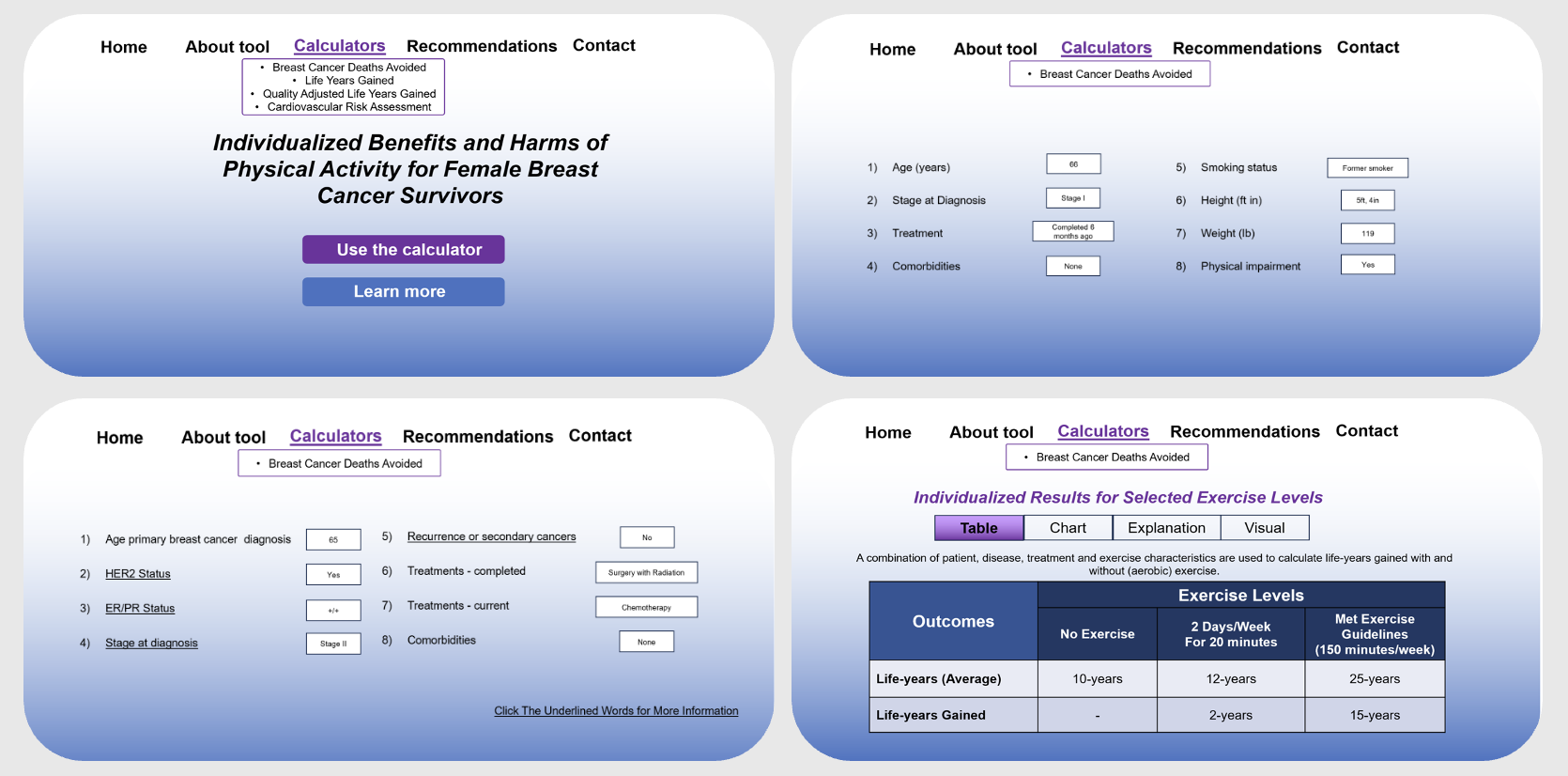


Supplemental Table 2. Survivor Characteristics

| **Characteristics** |  |  |
| --- | --- | --- |
| **Demographic** | n | % |
| ***Age*** (M (SD)) | 47.2 (12.2) | |
| <50 years | 65 | 66.3 |
| ≥50-<65 years | 17 | 17.3 |
| ≥65 years | 14 | 14.3 |
| Missing | 2 | 2.0 |
| ***Race and ethnicity*** |  |  |
| Alaska Native | 11 | 11.2 |
| American Indian | 10 | 10.2 |
| Asian | 10 | 10.2 |
| Black or African American | 10 | 10.2 |
| Hispanic | 22 | 22.4 |
| Middle Eastern and North African | 11 | 11.2 |
| Native Hawaiian | 10 | 10.2 |
| White | 14 | 14.3 |
| ***Sexual orientation*** |  |  |
| Bisexual | 3 | 3.1 |
| Lesbian | 7 | 7.1 |
| Straight | 85 | 86.7 |
| Missing | 3 | 3.1 |
| ***Disability status*** |  |  |
| No disability | 40 | 40.8 |
| Disability | 53 | 54.1 |
| Missing | 5 | 5.1 |
| **Exercise level** |  |  |
| Meet aerobic exercise guidelines (≥150min/week) | 77 | 77.4 |
| Meet muscle-strengthening exercise guidelines (≥2days/week) | 73 | 80.3 |
| **Clinical characteristics** |  |  |
| ***Years since diagnosis*** (M (SD)) | 13.0 (15.0) | |
| ***Diagnosed with another cancer (excluding skin cancer) other than breast cancer?*** | | |
| No - never been diagnosed | 73 | 74.5 |
| Yes - before being diagnosed with breast cancer | 8 | 8.2 |
| Yes - after being diagnosed with breast cancer | 17 | 17.3 |
| **Recurrence** |  |  |
| None of the above | 33 | 33.7 |
| Local recurrence | 28 | 28.6 |
| Regional recurrence | 18 | 18.4 |
| Distant recurrence | 16 | 16.3 |
| Contralateral breast cancer or secondary breast cancer | 6 | 6.1 |
| Another type of cancer | 36 | 36.7 |
| ***Survivorship stage*** |  |  |
| Pre-treatment | 3 | 3.1 |
| On-treatment | 47 | 48.0 |
| Post-treatment | 44 | 44.9 |
| Palliative care | 2 | 2.0 |
| Unknown | 2 | 2.0 |
| ***Stage of diagnosis*** |  |  |
| Stage 0 (ductal carcinoma in-situ (DCIS)) | 4 | 4.1 |
| Stage I (localized, has not spread) | 29 | 29.6 |
| Stage II (localized/regional) | 31 | 31.6 |
| Stage III (regional) | 20 | 20.4 |
| Stage IV (distant/metastatic) | 12 | 12.2 |
| Unsure | 0 | 0.0 |
| Unknown | 2 | 2.0 |
| ***Hormone receptor status*** |  |  |
| ER+/PR+ | 32 | 32.7 |
| ER-/PR- | 24 | 24.5 |
| ER+/PR- | 21 | 21.4 |
| ER-/PR+ | 7 | 7.1 |
| Unsure | 12 | 12.2 |
| Unknown | 2 | 2.0 |
| ***HER2 status*** |  |  |
| HER2-Positive | 34 | 34.7 |
| HER2-Negative | 43 | 43.9 |
| Unsure | 19 | 19.0 |
| Unknown | 2 | 2.0 |
| ***Conditions*** |  |  |
| Ataxia | 17 | 17.3 |
| Bone metastases | 12 | 12.2 |
| Extreme fatigue | 46 | 46.9 |
| Lymphedema | 33 | 33.7 |
| Peripheral neuropathy | 30 | 30.6 |
| None of the above | 20 | 20.4 |
| ***Chemotherapy*** |  |  |
| Never received treatment | 21 | 21.4 |
| Currently receiving treatment | 18 | 18.4 |
| Completed treatment | 54 | 55.1 |
| Unknown | 5 | 5.1 |
| ***Hormone therapy*** |  |  |
| Never received treatment | 23 | 23.5 |
| Currently receiving treatment | 26 | 26.5 |
| Completed treatment | 43 | 43.9 |
| Unknown | 6 | 6.1 |
| ***Immunotherapy*** |  |  |
| Never received treatment | 40 | 40.8 |
| Currently receiving treatment | 32 | 32.7 |
| Completed treatment | 20 | 20.4 |
| Unknown | 6 | 6.1 |
| ***Radiation*** |  |  |
| Never received treatment | 23 | 23.5 |
| Currently receiving treatment | 26 | 26.5 |
| Completed treatment | 43 | 43.9 |
| Unknown | 6 | 6.1 |
| ***Surgery*** |  |  |
| Never received treatment | 11 | 11.2 |
| Currently receiving treatment | 12 | 12.2 |
| Completed treatment | 65 | 66.3 |
| Do not know | 10 | 10.2 |
| ***Targeted therapy*** |  |  |
| Never received treatment | 39 | 39.8 |
| Currently receiving treatment | 29 | 29.6 |
| Completed treatment | 26 | 26.5 |
| Do not know | 4 | 4.1 |
| **Contextual characteristics** |  |  |
| ***Region*** |  |  |
| Midwest | 17 | 17.3 |
| Northeast | 13 | 13.3 |
| South | 40 | 40.8 |
| West | 26 | 26.5 |
| Missing | 2 | 2.0 |
| ***Urbanicity/rurality*** |  |  |
| Urban | 42 | 42.9 |
| Suburban | 43 | 43.9 |
| Rural | 11 | 11.2 |
| Missing | 2 | 2.0 |
| ***Living arrangements*** |  |  |
| Live in a house/apartment that you own | 54 | 55.1 |
| Live in a house/apartment that you rent | 23 | 23.5 |
| Live rent-free with family or friends | 15 | 15.3 |
| Refused | 1 | 1.0 |
| Missing | 5 | 5.1 |
| **Relationship status** |  |  |
| Married or living as married | 65 | 67.7 |
| Unmarried or not living as married | 30 | 31.3 |
| Missing | 3 | 3.0 |
| ***Education*** |  |  |
| ≤High School | 22 | 22.4 |
| Some college | 30 | 30.6 |
| ≥Bachelors degree | 42 | 42.9 |
| Missing | 4 | 4.1 |
| ***Household income*** |  |  |
| <$35,000 | 16 | 16.3 |
| ≥$35,000-<$75,000 | 30 | 30.6 |
| ≥$75,000 | 45 | 45.9 |
| Refused | 1 | 1.0 |
| Missing | 6 | 6.1 |
| ***Employment*** |  |  |
| Working full-time (35+ hours/week) | 59 | 60.2 |
| Working part-time (1-34 hours/week) | 11 | 11.2 |
| Retired | 11 | 11.2 |
| Raising children full-time, full-time caregiver, or keeping house | 3 | 3.1 |
| Disabled, permanently or temporarily | 6 | 6.1 |
| Other/Missing | 8 | 8.2 |

Supplemental Table 3. Breast cancer survivor agreement that exercise benefits should be included in a tool

| **Benefit** | **Agreement** | **95% CI** | |
| --- | --- | --- | --- |
|  |  | **Lower** | **Upper** |
|  | **%** | | |
| Greater ability to do everyday tasks | 74.0 | 63.8 | 84.2 |
| Improved quality of life | 72.6 | 62.1 | 83.2 |
| Less tiredness / more energy | 71.4 | 60.8 | 82.0 |
| Improved bone health | 71.1 | 60.4 | 81.8 |
| Improved sleep | 71.1 | 60.4 | 81.8 |
| Less likely to have the cancer come back | 70.8 | 60.0 | 81.6 |
| Helps you live longer | 68.4 | 57.1 | 79.7 |
| Less like to experience adverse side effects during treatment | 66.3 | 54.8 | 77.8 |
| Less likely to die of breast cancer | 65.3 | 53.6 | 77.0 |
| Less likely to die of cardiovascular disease (heart disease) | 63.3 | 51.3 | 75.3 |
| Less likely to experience anxiety | 62.2 | 50.1 | 74.4 |
| Less likely to experience lymphedema (swelling) | 62.2 | 50.1 | 74.4 |
| Less likely to experience depressive symptoms | 61.9 | 49.6 | 74.1 |
| Less likely to die of all causes | 58.8 | 46.0 | 71.5 |
| Less likely to be admitted to hospital | 58.8 | 46.0 | 71.5 |
| Improved cognition (memory) | 55.2 | 41.8 | 68.6 |

Note. Responses of “agree” or “strongly agree” were considered as agreement

Supplemental Table 4. Breast cancer survivor agreement that conditions associated with breast cancer or related treatment should be included in a tool

| **Condition** | **Agreement** | **95% CI** | |
| --- | --- | --- | --- |
|  |  | **Lower** | **Upper** |
|  | **%** | | |
| Cognitive difficulty (mental slowing) | 75.5 | 65.7 | 85.3 |
| Slowing and fatigue | 66.3 | 54.8 | 77.8 |
| Ataxia (problems with coordination, balance, walking) | 66.3 | 54.8 | 77.8 |
| Lymphedema (swelling due to build-up of lymph fluid in the body) | 63.9 | 52.0 | 75.9 |
| Sarcopenia/muscle weakness | 62.5 | 50.3 | 74.8 |
| Cardiopulmonary disease | 61.9 | 49.6 | 74.1 |
| Neuropathy (weakness, numbness, and pain) | 61.2 | 48.9 | 73.6 |
| Symptom clusters (symptoms that occur at the same time) | 60.8 | 48.4 | 73.3 |
| Arthritis/musculoskeletal issues | 59.8 | 47.2 | 72.4 |
| Bone loss | 59.4 | 46.6 | 72.1 |
| Nausea or diarrhea | 58.3 | 45.4 | 71.2 |
| Severe nutritional deficiencies (e.g., low calcium) | 58.2 | 45.4 | 71.0 |
| Bone metastases | 52.6 | 38.8 | 66.5 |
| Sexual dysfunction | 50.5 | 36.5 | 64.5 |

Supplemental Table 5. Breast cancer survivor agreement that demographic, clinical, and contextual characteristics should be included as inputs in a tool

|  | **Agreement** | **95% CI** | |
| --- | --- | --- | --- |
|  |  | **Lower** | **Upper** |
|  | % | | |
| Home exercise resources (access to exercise resources at home) | 79.6% | 70.6% | 88.5% |
| Readiness to exercise | 73.5% | 63.3% | 83.7% |
| Age | 72.2% | 61.7% | 82.7% |
| Historical treatment | 68.0% | 56.8% | 79.3% |
| Current comorbidities (e.g., diabetes, hypertension) | 68.0% | 56.8% | 79.3% |
| Food (access to healthy food) | 67.3% | 56.1% | 78.6% |
| Current treatment | 66.3% | 54.8% | 77.8% |
| Childcare (child supervision) | 65.3% | 53.6% | 77.0% |
| Exercise facility/resource access (access to facility or exercise program in the neighborhood or at work) | 65.3% | 53.6% | 77.0% |
| Stage at diagnosis (e.g., stage I/localized) | 63.9% | 52.0% | 75.9% |
| Physical impairments (e.g., mobility, reaching) | 60.8% | 48.4% | 73.3% |
| Psychological impairments (e.g. changes in mood, feeling down) | 60.8% | 48.4% | 73.3% |
| Cognitive impairments (e.g., changes in memory or attention, difficulty problem solving) | 58.8% | 46.0% | 71.5% |
| Transport (availability of transportation) | 58.2% | 45.4% | 71.0% |
| Neighborhood safety (safety of local environment) | 58.2% | 45.4% | 71.0% |
| Functional impairments (e.g., challenges with daily living) | 57.7% | 44.8% | 70.7% |
| Patient exercise preferences (e.g., what activities survivors enjoy doing) | 56.7% | 43.6% | 69.8% |
| Financial problems (problems with insurance or debt) | 55.1% | 41.8% | 68.4% |
| Tumor characteristics (e.g., tumor grade, ER/PR status) | 54.6% | 41.2% | 68.0% |
| Phone (availability of a phone to connect with peer and healthcare professional) | 52.0% | 38.3% | 65.8% |
| Clothing (availability of appropriate clothing and shoes) | 51.5% | 37.7% | 65.4% |
| Internet (availability of internet to check email and schedule exercise sessions) | 51.0% | 37.2% | 64.9% |
| Housing (availability of safe and secure housing) | 50.5% | 36.5% | 64.5% |
| Race and ethnicity | 43.6% | 28.4% | 58.8% |
| Utilities (had utility companies cut off service due to not paying bills) | 41.8% | 26.7% | 56.9% |
| Residential greenness (availability of green spaces) | 41.2% | 26.0% | 56.5% |

Supplemental Table 6. Other inputs survivors commented that should be included as inputs in a tool (n=63)

| **Suggested inputs** | n | % |
| --- | --- | --- |
| Physical limitations* | 8 | 12.9 |
| Tool usability/accessibility* | 8 | 12.9 |
| Social support/network | 6 | 9.7 |
| Budget/finances/income* | 6 | 9.7 |
| Age* | 3 | 4.8 |
| Medical history | 3 | 4.8 |
| Mental health (e.g., anxiety, stress, depression) | 3 | 4.8 |
| Tracking devices | 3 | 4.8 |
| Safety/security | 2 | 3.2 |
| Nutrition* | 2 | 3.2 |
| Work or personal obligations | 2 | 3.2 |
| Exercise history | 2 | 3.2 |
| Culture | 1 | 1.6 |
| Location | 1 | 1.6 |

Notes. Though the question pertained to inputs, survivors still commented on tool usability/accessibility; *Survivors mentioned inputs that were measured by survey items
